# The impact of specialist palliative care on healthcare utilization among patients with breast cancer: a nationwide register-based cohort study

**DOI:** 10.1007/s12282-025-01759-7

**Published:** 2025-08-14

**Authors:** Nelli-Sofia Nåhls, Timo Carpén, Mikko Nuutinen, Tiina Saarto

**Affiliations:** 1 Department of Oncology, Vaasa Central Hospital, The Wellbeing Services County of Ostrobothnia, Vaasa, Finland; 2 Department of Oncology, Comprehensive Cancer Centre, University of Helsinki, Helsinki, Finland; 3 Palliative Care Center, Comprehensive Cancer Center, Helsinki University Hospital, Faculty of Medicine, University of Helsinki, Helsinki, Finland; 4 Nordic Healthcare Group, Helsinki, Finland

**Keywords:** Specialist palliative care, Breast cancer, Emergency department, Healthcare utilization, End-of-life care

## Abstract

**Background:**

The incidence of breast cancer and the related mortality in Finland are among the highest in the world. Specialist palliative care (SPC) has been shown to improve quality of life and potentially reduce intensive resource use at the end of life among patients with advanced cancer. We aimed to perform a nationwide evaluation of the timing of the first SPC contact and its impact on hospital resource utilization in patients with breast cancer.

**Methods:**

A nationwide retrospective cohort analysis included 881 breast cancer patients who died in Finland in 2019, with data drawn from national registries. Patients were divided into two groups according to the time of their first SPC contact: Group I (> 30 days before death) and Group II (≤ 30 days before death or no SPC contact).

**Results:**

SPC contact was established for 288 (35%) patients, with a median interval of 89 days from initial SPC contact to death. During the last month of life, patients in Group I had fewer emergency department contacts (46% vs. 58%, p = 0.004) and fewer hospitalizations in secondary care (28% vs. 48%, p < 0.001), compared patients in Group II. Additionally, patients in Group I utilized hospital-at-home services more often (42% vs. 7%, p < 0.001) and had a higher likelihood of dying in SPC wards (15% vs. 3%, p < 0.001) rather than in hospital (65% vs. 77%, p < 0.001).

**Conclusion:**

Timely SPC contact was associated with fewer acute hospital contacts and a reduced likelihood of hospital death, underscoring the importance of timely palliative care integration for patients with advanced breast cancer.

## Introduction

Breast cancer is the most frequently diagnosed malignancy among women worldwide, with an estimated 2.3 million new cases in 2020 [1]. In Finland, it accounts for approximately 30% of all cancers in women, with an incidence rate of 173 per 100,000 in 2022 and about 800 deaths annually [2]. Despite advances in detection and treatment, breast cancer remains a major cause of cancer-related mortality [3], underscoring the need for effective end-of-life (EOL) care strategies.

Specialist palliative care (SPC) is a multidisciplinary approach that focuses on symptom relief, psychosocial support, and quality of life (QOL) improvement for patients with life-limiting illnesses [4]. A growing body of evidence suggests that the early integration of palliative care into standard oncologic treatment leads to improved QOL and less aggressive EOL care [5–7]. For instance, Scibetta et al. [8] showed that earlier palliative care consultations were associated with fewer emergency department visits, hospital admissions, and intensive care unit stays in the final month of life. Similarly, findings from Seow et al. [9] indicate that earlier palliative care engagement significantly reduces hospitalizations in the last month of life of patients with cancer.

Although most existing data are derived from broader cancer populations, emerging evidence in metastatic breast cancer suggests similar benefits from timely palliative care integration. For instance, integrated SPC services in this subgroup have been linked to reductions in aggressive EOL interventions [10, 11]. Schmitz et al. [12] also reported high healthcare utilization during the last six months of life among patients with advanced breast cancer, underscoring the potential value of timely SPC referral. However, national-level data examining how the timing of SPC influences hospital-based care and place of death in this population remain limited.

The final month of life is widely recognized as the most intensive period of healthcare utilization in cancer care. During this time, patients are at increased risk of emergency department visits, hospitalizations, and other acute care interventions, often reflecting aggressive and potentially avoidable care near the end of life [13–15]. While palliative care has been shown to reduce such burdens, there is no consensus regarding the optimal timing of SPC initiation to achieve these benefits [8, 9]. Recent registry-based evidence has supported the use of the final 30 days of life as a clinically meaningful window for evaluating the impact of SPC on healthcare resource utilization [16].

Building on this foundation, our nationwide retrospective cohort study aimed to examine whether the timing of SPC contact initiated more than 30 days before death is associated with reduced acute hospital resource use and improved access to palliative care services during the final month of life among patients with breast cancer in Finland.

## Patients and methods

### Study design and setting

We conducted a nationwide, retrospective cohort study in Finland, including all patients who died of breast cancer in 2019. Patients were identified from the 2019 Causes of Death Register (Statistics Finland) using the International Classification of Diseases, 10th Revision (ICD-10) codes for malignant neoplasm of the breast. Data were collected from the beginning of 2018 until the end of 2019. The total number of patients fulfilling the criteria was 882. Finally, 881 patients were included as one patient who died outside of Finland was excluded from the study. The study was reported in accordance with the Strengthening the Reporting of Observational Studies in Epidemiology (STROBE) guidelines [17].

### Data collection

Information was retrieved from two national data repositories: the National Care Register, which compiles records of healthcare contacts from both public and private providers, and Kanta Services, a legally mandated digital platform encompassing data from social welfare and healthcare sectors. By linking these databases using a health service unit code list, it was possible to gather sociodemographic data and details on healthcare utilization, social care services, and SPC services. Further information on data collection processes has been published previously [18–20].

### Utilization of health and social care services

Primary healthcare in Finland is delivered by municipal health centers staffed by general practitioners, while secondary care is provided at 20 hospital district hospitals and tertiary care at five university hospitals. For the purposes of this analysis, secondary and tertiary care are combined and collectively referred to as “secondary care.” The dataset included records on outpatient clinic contacts (both primary and secondary care), hospital admissions, emergency department contacts, social care services, and home care typically provided by practical nurses.

### Specialist palliative care contact

Palliative care in Finland is organized at both general and specialist levels. In this study, SPC was defined as care delivered by palliative care specialists in outpatient clinics, hospital-at-home services, specialized palliative care inpatient wards or hospices, and through inpatient consultations. An SPC contact was recorded at the time of the first documented interaction with any of these specialist services. Each encounter, whether an outpatient visit, inpatient consultation, or admission to a specialized ward, counted as one SPC contact. Patients were allocated to two groups based on the timing of their first SPC contact. Those whose initial SPC contact occurred more than 30 days before death were classified as Group I, whereas patients with no SPC contact or whose first SPC contact took place 30 days or fewer before death were placed in Group II. Based on previous studies, the selected groups were determined by evidence indicating that periods shorter than one month are insufficient to achieve the benefits associated with palliative care [16, 21].

From the data, ICD-10 code Z51.5 was collected, which indicates a palliative care decision, i.e. a situation in which curative or disease-modifying treatments are discontinued, and the focus shifts primarily toward symptom management and QOL.

### Place of death

Information on the place of death was derived from the 2019 Causes of Death Register. The data permitted classification of death as occurring at home, in a long-term care facility, in a hospital (comprising both primary and secondary care hospitals), or in a specialized palliative care ward. The last category allowed identification of patients who died under dedicated palliative care services.

### Ethical statement

The study was performed with the Finnish Institute for Health and Welfare (THL) as part of the Project on Quality Information on Palliative Care and End-of-life Care. The study was approved by THL Dnr: 12345556. According to the Finnish legislation for research, no separate ethics committee approval was needed, as data used in the study concerned deceased patients.

### Statistics

All statistical analyses were performed using IBM-SPSS version 29 (IBM Corp, Armonk, NY, USA). Descriptive statistics are reported as medians and ranges, numbers of incidences and percentages. The groups were categorized according to the timing of the first SPC contact. Pearson’s chi-squared test and Fisher exact tests were used to compare categorical variables. Comparisons between hospital days in different groups were done using Mann–Whitney test as the distributions were not equal. Logistic regression analyses were performed to identify independent factors associated with emergency department contacts and hospitalizations during the last 30 days of life. Variables included in the multivariable models were age, municipality type (categorized as urban, semi-urban, rural), and timing of SPC contact. A p value of < 0.05 was considered statistically significant.

## Results

### Patient characteristics

**Fig. 1:**
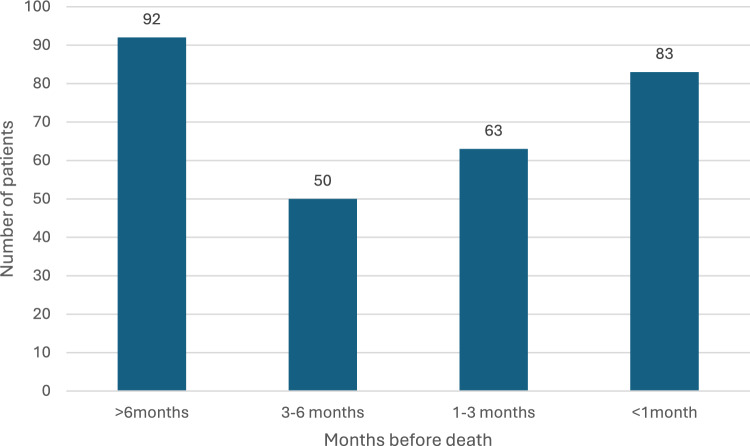
Timing of first contact with specialist palliative care (SPC) in relation to death

The total study cohort comprised 881 patients, with demographic and clinical information shown in Table 1. The mean age at death was 73 years, and nearly all patients (99%, n = 874) were female. SPC contact was established in 288 patients (35%), with 205 (25%) receiving their first SPC contact more than 30 days before death. The median time from the first SPC contact to death was 89 days (range 0–723), with 153 days in Group I (range 31–723) and 12 days in Group II (range 0–30). Timing of SPC contact in relation to death is shown in Fig. 1. ICD-10 code Z51.5 was recorded for half of the patients (55%) and was more frequent in Group I (77% vs. 49%, p < 0.001).

**Table 1:** Patient characteristics

Number of patients (%)	All patients (n = 881)	Group I (n = 205)	Group II (n = 676)	P-value
Age in years median (range)	74 (28–100)	74 (30–99)	74 (28–100)	0.830
*Municipality type*				
Urban	631 (72%)	173 (84%)	458 (68%)	< 0.001
Semi-urban	138 (16%)	25 (12%)	113 (17%)	0.119
Rural	111 (13%)	7 (3%)	104 (15%)	< 0.001
ICD-10 Diagnosis code Z51.5 Palliative care	487 (55%)	157 (77%)	330 (49%)	< 0.001
SPC contact	288 (35%)	205 (100%)	83 (12%)	< 0.001
Median time from the first SPC contact to the death	89 days (0–723 days)	153 days (31–723 days)	12 days (0–30 days)	< 0.001

*n* number of patients, *SPC* specialist palliative care

Group I = first SPC contact > 30 days before death, Group II = first SPC contact ≤ 30 days before death or no contact

### Healthcare utilization in the last 30 days of life

Substantial differences in healthcare service use emerged between patients in Group I and II. Table 2 summarizes healthcare resource utilization and place of death within the last 30 days of life. More than half of the patients (55%) had contacts with the emergency department, but patients in Group I had significantly fewer emergency department contacts compared to those in Group II (46% vs. 58%, p = 0.004). Overall hospitalizations were also less frequent patients in Group I (65% vs. 79%, p < 0.001). In the multivariable logistic regression model for emergency department contacts (Table 3), younger age (p < 0.001), municipality type (p = 0.001), and initiation of specialist palliative care within the last 30 days of life or not at all (p < 0.001) were significantly associated with higher odds of contact. Patients living in semi-urban and rural areas had significantly fewer emergency department contacts compared to those in urban areas. For hospitalizations (Table 4), both younger age (p < 0.001) and SPC initiated within 30 days of death or not at all (p < 0.001) were associated with an increased risk of hospitalization.

**Table 2:** The impact of timing of first specialist palliative care contact on the use of hospital services and place of death in the last 30 days of life

Number of patients (%)	All patients (n = 881)	Group I (n = 205)	Group II (n = 676)	P-value
Emergency department contacts	486 (55%)	95 (46%)	391 (58%)	0.004
*Outpatient clinic contacts*				
Secondary care	466 (53%)	68 (33%)	398 (59%)	< 0.001
Primary health care	567 (64%)	115 (56%)	452 (67%)	0.005
*Hospitalizations*				
All hospitalizations	665 (76%)	133 (65%)	532 (79%)	< 0.001
Secondary care	381 (43%)	58 (28%)	323 (48%)	< 0.001
Primary health care	468 (53%)	107 (52%)	361 (53%)	0.762
*Specialist palliative care*				
Palliative care outpatient unit	61 (7%)	37 (18%)	24 (4%)	< 0.001
Hospital-at-home	134 (15%)	87 (42%)	47 (7%)	< 0.001
Special palliative care ward	58 (7%)	33 (16%)	25 (4%)	< 0.001
Social services	117 (13%)	25 (12%)	92 (14%)	0.601
Home care	379 (43%)	101 (49%)	278 (41%)	0.039
*Place of death*				
Home	93 (11%)	27 (13%)	66 (10%)	0.164
Hospital	652 (74%)	133 (65%)	519 (77%)	< 0.001
Long term care	84 (10%)	15 (7%)	69 (10%)	0.217
Specialist palliative care ward	52 (6%)	30 (15%)	22 (3%)	< 0.001

*n* number of patients, *SPC* specialist palliative care

Group I = first SPC contact > 30 days before death, Group II = first SPC contact ≤ 30 days before death or no contact

**Table 3:** Factors associated with emergency department contacts during the last 30 days of life: logistic regression analysis

	n	OR	95% CI	p-value
Age at death	881	0.974	0.964–0.985	< 0.001
*Municipality type*				0.001
Urban	631	Reference		
Semi-urban	138	0.571	0.391–0.836	
Rural	111	0.565	0.372–0.859	
*Timing of specialist palliative care contact*				< 0.001
Group I	205	Reference		
Group II	676	1.775	1.281–2.460	

*n* number of patients, *SPC* specialist palliative care, *OR* odds ratio, *CI* confidence interval

^†^Age at death was analyzed as a continuous variable. Group I = first SPC contact > 30 days before death, Group II = first SPC contact ≤ 30 days before death or no contact

**Table 4:** Factors associated with hospitalizations during the last 30 days of life: logistic regression analysis

	n	OR	95% CI	p-value
Age at death	881	0.975	0.963–0.988	< 0.001
*Municipality type*				0.782
Urban	631	Reference		
Semi-urban	138	1.146	0.730–1.801	
Rural	111	0.934	0.573–1.523	
*Timing of specialist palliative care contact*				< 0.001
Group I	205	Reference		
Group II	676	2.029	1.428–2.884	

*n* number of patients, *SPC* specialist palliative care, *OR* odds ratio, *CI* confidence interval

^†^Age at death was analyzed as a continuous variable. Group I = first SPC contact > 30 days before death, Group II = first SPC contact ≤ 30 days before death or no contact

**Fig. 2:**
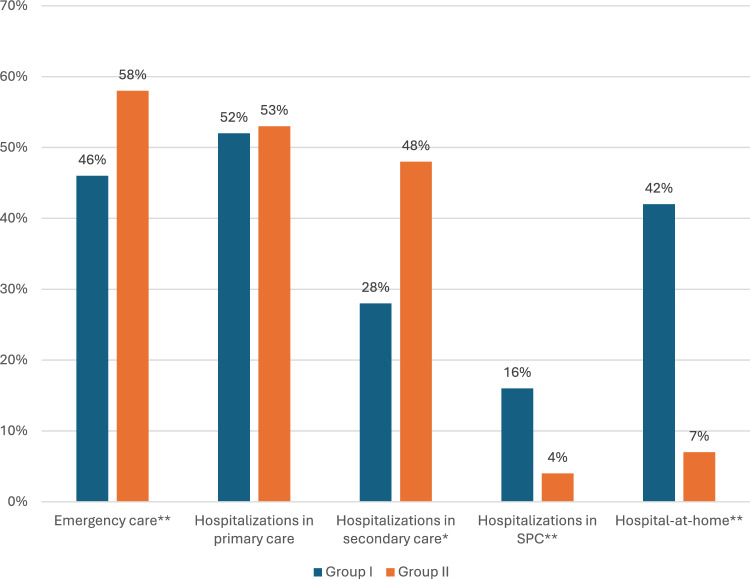
Emergency department contacts and hospitalizations in the last month of life according to the time of first SPC contact. *p-value < 0.05 and **p-value < 0.001

Patients in Group I received hospital-at-home care in the final month of life more often than those in Group II (42% vs. 7%, p < 0.001) (Fig. 2).

### Use of hospital-at-home services

Patients who received hospital-at-home services (compared to those without such service) had fewer hospital stays (64% vs. 78%, p < 0.001), were more likely to receive home care (65% vs. 39%, p < 0.001), and had a higher likelihood of dying at home (23% vs. 8%, p < 0.001) or in a SPC ward (10% vs. 5%, p = 0.015), whereas hospital deaths were significantly less common (55% vs. 78%, p < 0.001).

### Place of death

Despite palliative care integration, the majority of patients (74%) of patients died in a hospital setting. However, patients in Group I were significantly less likely to die in hospital (65% vs. 77%, p < 0.001) and more likely to die in an SPC ward (15% vs. 3%, p < 0.001) compared to those in Group II.

## Discussion

This nationwide retrospective cohort study demonstrates significant associations between the timing of SPC initiation and healthcare utilization among patients with breast cancer in Finland during the EOL period. Our findings show that initiating SPC more than 30 days before death was associated with significantly lower use of acute hospital services during the final month of life, including fewer emergency department visits and hospitalizations. Early SPC involvement also correlated with increased use of hospital-at-home services and a greater likelihood of dying in a dedicated SPC ward.

The optimal timing for initiating SPC remains debated and varies considerably across existing studies [5–8]. However, our choice to use a 30-day threshold was guided by established EOL quality indicators [13–15] and recent evidence highlighting that healthcare utilization typically intensifies during the last month of life [16], a finding consistent across different patient groups, including both malignant and non-malignant diseases [20].

Consistent with existing literature [22], we found that SPC remains underutilized, with only 35% of patients having any documented SPC contact and a median interval from first SPC contact to death of 89 days. This aligns with international reports suggesting persistent delays in palliative care referrals across cancer types [11, 23]. For example, Lucchi et al. [11] demonstrated that only a minority of patients with metastatic breast cancer received timely palliative care referrals, resulting in a high proportion of hospital deaths. Similarly, Hausner et al. [23] highlighted improvements in earlier SPC referrals following clear evidence supporting early integration, though delays remain common. These previous studies, along with others [5–7], have consistently shown that early SPC contact improves symptom control, enhances QOL, and reduces intensive healthcare utilization at the EOL. However, to our knowledge, our study is the first nationwide analysis specifically demonstrating the impact of SPC timing on healthcare utilization and place of death among patients with breast cancer.

Healthcare utilization in our cohort remained high, reflecting the significant symptom burden and disease progression typical of advanced breast cancer [11, 12]. However, patients in Group I had markedly fewer emergency department contacts and hospitalizations during their final month of life. These results suggest that earlier SPC involvement facilitates proactive care planning and symptom control, thereby minimizing unnecessary acute care needs [8, 10, 24]. Importantly, even after adjusting for age and municipality type, earlier SPC contact remained an independent and clinically meaningful factor associated with reduced intensity of end-of-life care.

Hospital-at-home services were used much more frequently among patients with breast cancer who were in Group I compared to those in Group II. In addition, those who received hospital-at-home service tended to have fewer hospital admissions, were more likely to receive concurrent home care, and more commonly spent their final days outside the hospital – either at home or in an SPC ward. These observations are consistent with earlier research demonstrating that hospital-at-home models can reduce hospital utilization, improve satisfaction, and increase the likelihood of dying at home [25–27].

Many patients with advanced cancer express a wish to spend their final days at home; however, their actual place of death often does not align with these preferences [28, 29]. In a nationwide multicenter study conducted in France by Lucchi et al. [11], only 16.7% of patients with metastatic breast cancer died at home, while 63.3% died in the hospital and 20% in a palliative care unit—highlighting the gap between patient desires and real-world outcomes. In our cohort, 74% of patients died in the hospital, closely mirroring the high rate of hospital deaths observed in Lucchi et al. [11]. Nevertheless, patients in Group I were significantly more likely to die in an SPC ward (15% vs. 3%), suggesting that timely referral can help ensure access to expert symptom management and holistic support in a dedicated palliative setting.

The study’s strengths include its nationwide coverage, capturing all breast cancer deaths in Finland during 2019, ensuring high external validity within the Finnish healthcare system. Nevertheless, certain limitations must be acknowledged. Due to its retrospective design, causality cannot be conclusively determined. Although age and municipality type were adjusted for in multivariable logistic regression models, potential unmeasured confounders such as disease severity, socioeconomic status, and comorbidities might still have influenced the findings. Additionally, our study did not include patient-reported outcomes or caregiver experiences, essential components of comprehensive palliative care evaluations. Finally, the generalizability of our results outside Finland may be limited due to differences in healthcare infrastructure, palliative care accessibility, and cultural factors that influence care preferences. Despite these limitations, our findings provide critical real-world evidence supporting the timely integration of SPC into standard oncological practice.

## Conclusion

Our findings highlight the potential benefits of initiating SPC more than 30 days before death for breast cancer patients in terms of reduced acute healthcare utilization and increased likelihood of dying in an SPC setting. These results support the integration of timely SPC into the care pathway for patients with advanced breast cancer.

## Data Availability

The data generated during the current study are not publicly available as they are a part of a larger dataset owned by the Finnish Institute for Health and Welfare (THL). Data are however available from the principal author Tiina Saarto upon reasonable request and with permission of the THL.
